# Biocompatibility Testing for Implants: A Novel Tool for Selection and Characterization

**DOI:** 10.3390/ma16216881

**Published:** 2023-10-26

**Authors:** Walid Al-Zyoud, Dana Haddadin, Sameer Ahmad Hasan, Hussamaldeen Jaradat, Olfa Kanoun

**Affiliations:** 1 Department of Biomedical Engineering, School of Applied Medical Sciences, German Jordanian University, Amman 11180, Jordan; d.haddadin1@gju.edu.jo (D.H.); sameer.hasan@gju.edu.jo (S.A.H.); 2 Measurement and Sensor Technology, Chemnitz University of Technology, 09126 Chemnitz, Germany; hujar@hrz.tu-chemnitz.de

**Keywords:** biocompatibility, implants, mechanical property, health

## Abstract

This review article dives into the complex world of biocompatibility testing: chemical, mechanical, and biological characterization, including many elements of biocompatibility, such as definitions, descriptive examples, and the practical settings. The focus extends to evaluating standard documents obtained from reliable organizations; with a particular focus on open-source information, including FDA-USA, ISO 10933 series, and TÜV SÜD. We found a significant gap in this field: biomaterial scientists and those involved in the realm of medical device development in general, and implants in particular, lack access to a tool that reorganizes the process of selecting the appropriate biocompatibility test for the implant being examined. This work progressed through two key phases that aimed to provide a solution to this gap. A straightforward “yes or no” flowchart was initially developed to guide biocompatibility testing decisions based on the previously accumulated information. Subsequently, the Python code was employed, generating a framework through targeted questions. This work reshapes biocompatibility evaluation, bridging theory and practical implementation. An integrated approach via a flowchart and the Python code empowers stakeholders to navigate biocompatibility testing effortlessly. To conclude, researchers are now better equipped for a safer, more effective implant development, propelling the field towards improved patient care and innovative progress.

## 1. Introduction

The term “biocompatibility” is formed from two roots: life and compatibility. Biocompatibility relates to how living hosts interact with their environment. Life is living, while harmony is a functioning balance. Biocompatibility is establishing an environment or product compatible with humans [1].

This definition is only a literal translation of the phrase and does not consider the term’s purpose or usage. Researchers have examined and debated this concept over the years. Charles Homcy coined the term “biocompatibility”. A foundational study resulted in today’s conceptualization for assessing whether materials can coexist peacefully, meaning “biocompatibility” [1]. This definition is only a literal translation of the phrase and does not consider the term’s purpose or usage. This is where the effort of researchers over the years comes into play, since the concept has been examined and debated to achieve an acceptable definition.

Williams’ definition of biocompatibility, which is the most used currently, comes into play: “The capacity of a material to operate with an adequate host reaction in a given application.” [2]. William’s concept of biocompatibility is limited, since it eliminates any negative responses. For example, if a living host is exposed to a material without affecting their health or well-being, it is classified as biocompatible. This approach eliminates possible circumstances with negative consequences, such as causing damage or injury to the living host. Additional considerations must be taken into consideration when developing the concept of biocompatibility. For example, what makes up suitable materials’ behavior, and what form of response should be expected in the host exposed to a material or substance? These questions remain unanswered and yield a significant space for interpretation [3].

Biocompatibility requires an understanding of materials interacting with biological tissue. Defining such interactions is a major step in developing safe and effective materials. We need to understand and evaluate a material’s behavior and interaction with the body to assess its safety and efficacy. Research in biocompatibility seeks to identify the mechanisms involved once a material is introduced into a body or living cell. By understanding these mechanisms, scientists can improve the design of biocompatible materials [4].

**Figure 1:**
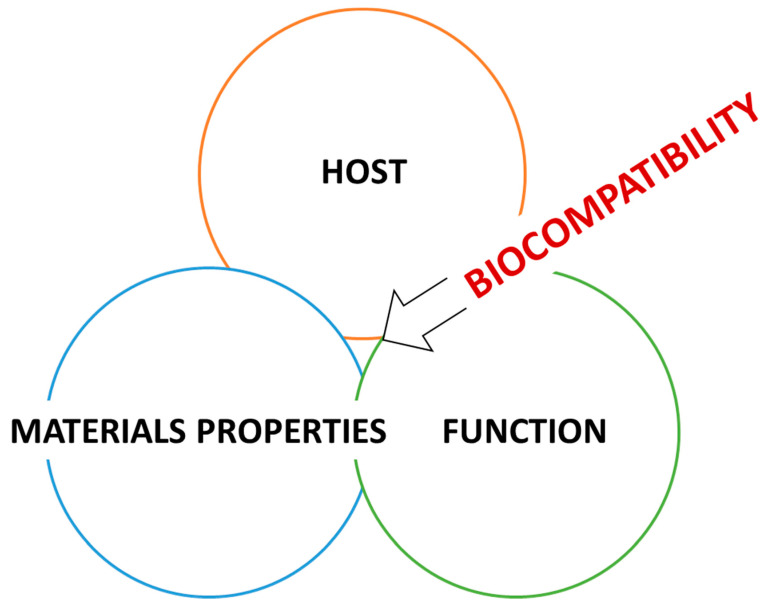
Biocompatibility is the relationship among the host, materials properties, and their functions.

Biocompatibility is a multidimensional concept that refers to the tissue and body interaction with several systems or conditions, such as chemical, metabolic, physiological, physical, and others. The central matter is associated with biomaterials’ contact with physiological tissues and understanding the consequences of these interactions, which is crucial to guarantee the safety and efficacy of the biomaterial. What happens after the procedure of inserting/implanting a material inside the human body for medical purposes can be considered a complicated process encompassing numerous routes engaged in the interaction of materials and human tissues (Figure 1). Understanding this relationship is critical in developing safe and biocompatible materials [2].

**Figure 2:**
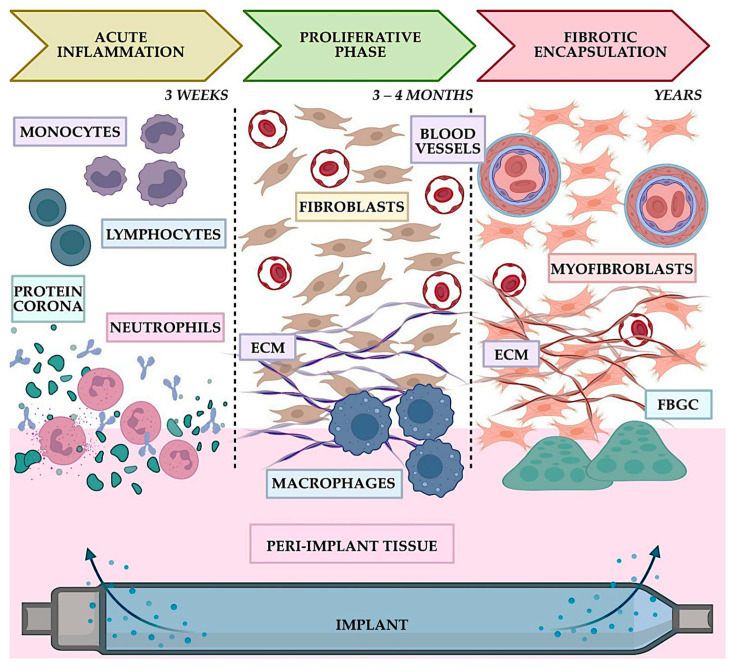
Typical timeline of the phases FBR to implants like an implantable drug delivery system modified from, adapted, and adopted from [6].

Materials–tissue interaction processes have been studied in many environments. Disruptions of natural physiological mechanisms within the body are likely caused by materials–tissue interactions. Both cellular and extracellular components must function normally. Deviations from normal behavior can weaken effectiveness, leading to failure and complications. The careful engineering and evaluation of materials and implants are necessary to ensure biocompatibility and safety for medical applications. Besides physical properties, biocompatibility is also determined by many factors. The kind and quality of the therapeutic action that puts the drug into touch with the tissues, for example, is crucial and is affected by a range of patient-related characteristics, such as age, gender, general health, concurrent disease, physical mobility, and lifestyle factors [2]. Human and animal cells take part in determining materials’ biocompatibility. This aspect is crucial, because the body can perceive the substance as foreign, potentially causing harm to tissues in the vicinity (Figure 2). Bringing a material into contact with tissue to fulfill medical procedures can harm the tissue and cause the local destruction of a segment of human tissues around the foreign body’s site [1,5].

Understanding biocompatibility’s underlying mechanisms helps researchers identify material properties that affect human body reactions [6]. Researchers often focus on two primary material properties: bulk material properties and surface material characteristics. Bulk material properties cover elasticity, shear strength, specific gravity, inertness, and the bulk modulus. Material surface characteristics include surface crystallinity, crystals preferred orientations, grain size, and wettability.

Using the term “biomaterial” is more logical when referring to healthcare-related materials. This includes materials used to fabricate various medical tools and devices, such as those used in implants and surgery procedures. Therefore, for a material to be referred to as a “biomaterial”, it must follow the definition assigned by The National Institute of Health Consensus Development Conference of November 1982, which states that “any substance (other than a drug) or combination of substances, synthetic or natural in origin, which can be used for any period of time, as a whole or as a part of a system which treats, augments, or replaces any tissue, organ, or function of the body” [7].

This review was motivated by the challenging selection process for biomaterials by scientists for specific applications. Implants may require tougher materials due to their permanent nature and the need for long-term support of the surrounding tissues. In such applications, the materials must be biocompatible, mechanically stable, and have the proper mechanical strength and stiffness to match the native tissues. Similarly, materials used to aid with organ functions, such as brain tissue regeneration or renal failure therapies, must have certain qualities that allow for the effective regeneration or replacement of damaged tissues. As a result, selecting materials for medical devices is considered a major challenge [7]. Biocompatibility has been discussed in this section, focusing on Williams’ definition. In the following section, an insight into the history of biocompatibility is reviewed.

## 2. History of Biocompatibility

Materials have been used to promote human health throughout history, with evidence extending back thousands of years before the common era. For example, the ancient Egyptians utilized copper and gold to make dental fillings, while the Romans used ivory to replace teeth. More advanced surgical procedure concepts have been developed since the nineteenth and twentieth centuries, which improved numerous individuals’ lives, resulting in the necessity for various biomaterials for medical applications, such as devices, implants, sutures, and prosthetic devices. It is worth noting that cellulose nitrate, while not commonly used in modern medical implants, has a historical significance in the development of implant materials. Its use in industries like photography and film provided valuable insights into the properties of polymers and their potential applications in the medical field. The knowledge gained from working with cellulose nitrate contributed to advancements and breakthroughs in developing materials used in medical implants [1].

The journey of understanding biomaterials and the concept of biocompatibility has been extensive. One example can be traced back to the late 1800s; a European specialist in Chicago endeavored to spare the life of a severely burned child by employing a unique approach to biocompatibility. The specialist transplanted skin from a living sheep onto the girl’s body, but unfortunately, she passed after some time. Despite the unsuccessful outcome, the specialist observed that the skin folds demonstrated the capability to nourish the child’s body, revealing the potential of the field of biocompatibility [8].

Another historical milestone was the effective utilization of celluloid to cure cranial anomalies, documented in a groundbreaking publication published in 1891. This transparent flammable plastic material played a significant role in inspiring professionals to utilize it in medical applications. This tremendous breakthrough in medical science cleared the path for the further research and development of implantable materials. This has resulted in modern medical implants comprising various materials, such as metals, ceramics, and polymers. These materials have been deliberately engineered to become biocompatible, enabling them to interact with human tissues safely and effectively without causing injury or unintended responses. The continuous development of novel materials and technology offers immense promise for the future of medical implants, potentially improving and saving countless lives [1].

Ilya Ilyich Metchnikoff, a prominent scientist credited with the discovery of macrophages and pioneering research on the destiny of implanted materials in live soft tissue, conducted a significant study on this subject in 1884. Metchnikoff’s groundbreaking investigation shed light on the biological mechanisms underlying the interaction between implanted materials and living tissue, significantly advancing our understanding in the field. His pivotal contributions have had a lasting impact on the scientific community and continue to shape contemporary research in this area. Metchnikoff’s observation on Starfish larvae proved his hypothesis about the biological process of a specific type of cells in the human body attacking any foreign body. These cells are now known as Macrophages. Metchnikoff stated that “I hypothesized that if my presumption was correct, a thorn introduced into the body of a starfish larva, devoid of blood vessels and nervous system, would have to be rapidly encircled by the motile cells, similarly to what happens to a human finger with a splinter.” This quote is an insightful explanation of his understanding of the topic, which triggered further research in this area [9].

Several surgeons experimented with prosthetic materials in the early 1900s. For example, the German physician Themistocles Glück used ivory and nickel-plated metal to create a hip prosthesis as early as 1891. The Czech surgeon Vitezlav Chlumsky also evaluated diverse types of joint interposition material over time but without understanding the toxicological or biocompatibility concerns. None of these trials would likely have been successful because of the lack of knowledge at the time about how implants should be designed and constructed [8].

A significant advancement in biocompatibility occurred with the discovery of ancient human bones in the state of Washington, estimated to be around 8000 years old. Upon examination, the remains revealed evidence of a spear wound and subsequent infection in the human pelvis. Remarkably, because of the absence of modern medical interventions during that era, the individual could have lived a long time before dying. Thus, scientists studied and analyzed the collagenous capsule surrounding the spear tip. This helped researchers to learn more about how ancient people treated injuries and understand their methods for healing them. In a separate discovery in 1931, a Mayan lady’s skull was discovered with three seashell dental implants. A radiological examination later revealed that these dental prostheses had been seamlessly integrated into the woman’s jawbone (osseointegrated). This shows that seashells were used as early as 600 BCE to replace teeth in humans [8].

In the realm of surgical advancements, the 1930s witnessed the introduction of glass balls for breast augmentation (mammoplasty) to enhance surgical outcomes. Moreover, an array of materials, including wood, leather, gold, rubber, magnesium, zinc, waxes, and plastics, were also experimented with during this era for similar purposes. During the same period, significant breakthroughs emerged with the commercial production of synthetic plastics, namely polyethylene and poly (methyl methacrylate) (PMMA), which found their application in surgical procedures. PMMA’s utility in cranioplasty was explored in scholarly discussions throughout the 1930s and 1940s. Dr. J. Bing’s influential research paper focused on PMMA’s behavior during surgery, offering a comprehensive understanding of its reactions. This pivotal article provided an exhaustive account of the potential side effects and risks associated with employing PMMA in skull reconstruction procedures [1].

During the 1940s, a breakthrough occurred in ophthalmology when British ophthalmologist Harold Ridley recognized the distressing eye injuries sustained by pilots due to shards of glass from broken windshields. To ease their pain and discomfort, Ridley investigated the suitability of poly (methyl methacrylate) (PMMA) for developing an intraocular lens (IOL)—the pioneering artificial lens implanted in humans. The belief in PMMA’s biocompatibility fueled its potential as a safe implant material. Ridley’s research supported this notion, as there have been no reported cases of adverse effects from PMMA lenses over fifty years later. Notably, the terms “biomaterial” and “biocompatibility” acquired prominence in scientific literature only in the late 1960s, when researchers delved into the compatibility of various materials with each other [8].

Table 1 reveals the history of medical research and materials. It illuminates the evolving understanding of biocompatibility among researchers and medical practitioners and their material selection processes. By examining the different fields and functions listed in the table, we understand the historical development and utilization of materials in the medical field. This information contributes to our knowledge of biocompatibility and the advancements made in medical practices throughout history.

## 3. Definition and Uses of Biocompatibility in Different Fields

**Figure 3:**
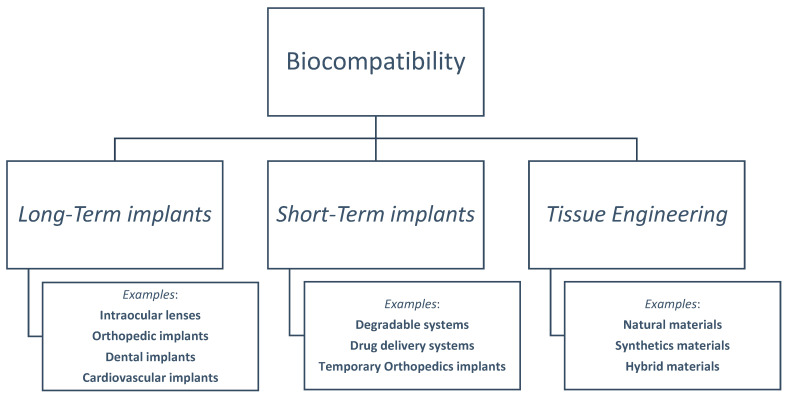
Flowchart that displays the subcategories of biocompatibility fields that follow the Williams Dictionary of Biomaterials.

Biomaterials and biocompatibility are intertwined in medical fields. We present an illustration of biocompatibility domains in Figure 3, based on the Williams Dictionary of Biomaterials. ISO and FDA documents may give different names for biocompatibility subcategories in later sections. These documents explain the reclassification of information and categories.

### 3.1. Long-Term Implants

The last decade saw a rise in long-term implant usage due to population growth, demand, and technology. In 2012, the number of Braenemark System implants administered worldwide was 7 million, and an additional million spinal rod implementations were conducted up to 2000 [20]. Long-term implants may include cardiovascular implants, intraocular lenses, orthopedics, and dental implants.

#### 3.1.1. Cardiovascular Implants

The mortality rate associated with cardiovascular diseases continues to be the highest in the world. In recent years, coronary artery disease (CAD) has caused a significant number of deaths in the country. Depending on the severity of the condition, it is possible to choose from a wide range of blood vessel therapies. An example of this is to insert a stent, performing an angioplasty, and, in cases of severe and widespread blocks (greater than 70%), performing a bypass graft operation [21]. This section discusses two major long-term cardiovascular implants: artificial heart valves and stents.

##### Artificial Heart Valves

**Figure 4:**
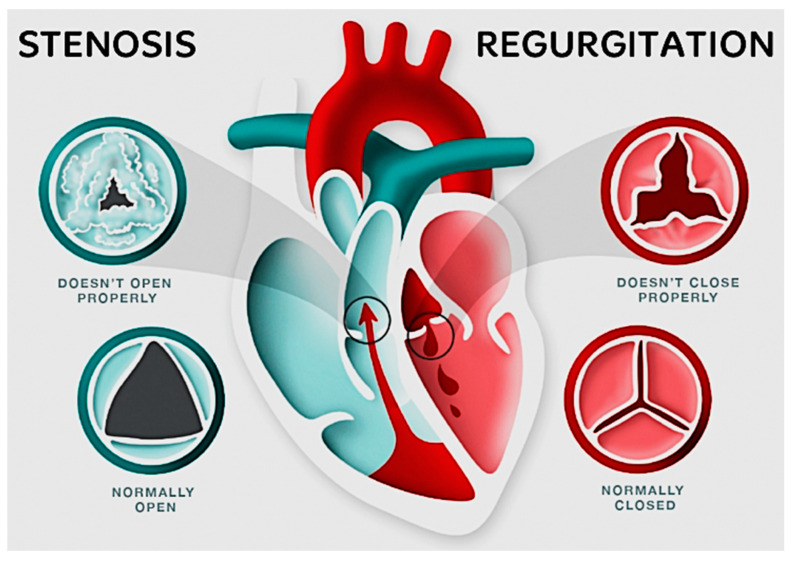
Top view of aortic and pulmonary valves in different cases of healthy and ill-functioning valves, adapted from [22].

Heart valve implants are required because of the important function of the four heart valves in the cardiovascular system. With each cardiac contraction, these valves work together to guarantee the unidirectional flow of blood. Disorders can harm the heart valves, causing issues like stenosis or regurgitation. Valve failures can happen because of disease or birth defects. Faulty valves can cause serious health issues like stroke and heart failure if left untreated. Damaged valves must be repaired or replaced for us to address these concerns. Surgical intervention provides two basic alternatives for valve replacement: mechanical valves constructed of artificial materials and tissue valves derived from biological sources [22]. Figure 4 below illustrates a basic example of how healthy and ill-functioning aortic and pulmonary valves appear when opened and closed [23].

**Figure 5:**
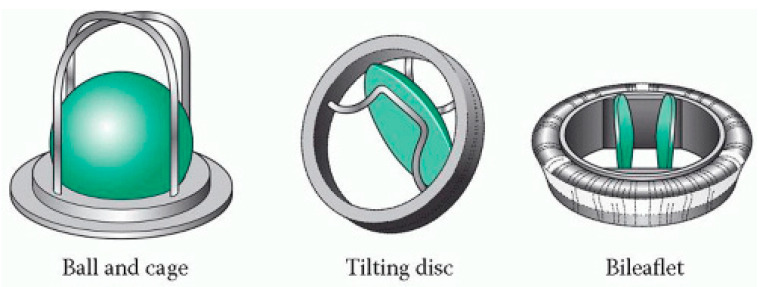
Some examples of mechanical heart valves, adapted from [25].

The synthetic materials used in a mechanical heart valve, like metal and synthetic polymers, are vital for cardiac surgery. There are two types of blood flow in artificial heart valves: central and lateral. A mechanical valve can be classified structurally into cage, spherical, disc, double lobe, and other categories [24]. Figure 5 illustrates the shape of a portion of these categories [25].

Because of their restricted biocompatibility, as evidenced by its inclination for blood clots to develop on its metal surfaces [22], patients typically have blood haemolysis, coagulation, and the requirement for anticoagulant medication. Because of their stability, heart surgeons frequently use them. Mechanical valves have three common elements: locking element, cover, and valve base. A brief description and examples of structures or materials used for these components are presented in Table 2 below [24].

For young people with a long-life expectancy who need a valve for a long time, the best choice is a mechanical valve. On the other hand, elderly patients with a limited life expectancy are better suited for tissue valves, which are made from biological tissues like the pericardium of pigs or cows. Patients who obtain tissue valves are less likely to need lifelong blood-thinning medication. This advantage arises from the lower risk of blood clots associated with tissue valves. However, one drawback to tissue valves is their tendency to deteriorate over time, as they are not as durable as mechanical valves. This could cause the need for a secondary or subsequent operation to replace the valve.

##### Stents

In cases of blood artery stenosis, cardiovascular stents are utilized to enhance blood flow. Angioplasty inserts coil-shaped stents into arteries to widen them. Stents are classified into two types: self-expanding stents composed of shape memory alloys such as Nitinol and stents placed in a catheter with a balloon made of 316L stainless steel. Stents can be categorized into four structural classes: mesh stents, tubular stents, ganglion-shaped stents, and annular coil stents. It is a critical characteristic of all stents that they suppress blood clot formation as they pass over their surface. It is important to avoid the formation of blood clots at the site of implantation, as this may cause arterial blockage. To prevent blood clotting, stents are coated with calcium phosphate or carbon. Resistance to blood pressure changes, a small diameter, flexibility, consistent cross-section under stress, high fatigue strength, clear route maintenance, compatibility with the body, resistance to infection, availability, and ease of implantation are all important concerns for stents [24].

#### 3.1.2. Intraocular Lenses

Intraocular lenses (IOLs) are a prime example of long-term implanted devices specifically designed to aid human vision and are implanted inside the human eye. Thus, IOLs’ materials must be physically compatible with the incubating tissue. In addition, it must have a high resistance to degradation to function in the long term [2].

The variations in materials utilized in these devices are attributed to the need for various chemical structures or surface properties to meet mechanical and physical properties such as flexibility, inertness, and regulating surface hydrophobicity/hydrophilicity [26]. Hydrophilicity refers to a material’s affinity towards water and the ability to maximize water contact [27]. Managing these properties is crucial for ensuring clinical usage and achieving the desired functionality [2]. Silicone stands out as a prominent example of a flexible material. Its malleability makes it an excellent choice for IOLs, as it maintains chemical stability and offers various mechanical properties. The biocompatibility of IOLs plays a crucial role in their overall implantation success [26].

#### 3.1.3. Orthopedics

Long-term orthopedic implants are another example of long-term implants. Materials for such applications must exhibit a remarkable resistance to corrosion and wear. In addition to their physical attributes, their chemical stability and appropriate microstructural properties are crucial considerations for orthopedic applications.

Ceramics, which are inorganic and nonmetallic materials, offer a diverse range of features suitable for various applications, particularly in hip and knee repairs, such as ceramics comprising a cobalt–chrome (Co-Cr) metal alloy.

Several orthopedic materials, such as polymers, resorbable materials, and metallic materials, have been utilized. Polymers, including acrylic resins, polyethylene, and others, are known for their structural stability, cost-effectiveness, and relative biocompatibility. This class of materials is suitable for anchoring or prosthesis applications and devices.

Resorbable biodegradable materials are a class of materials that serve therapeutic purposes, such as bone substitutes and fracture healing, for example, polyglycolide (PGA) and polylactide (PLA).

Metallic materials are known for their excellent mechanical properties and are commonly utilized in prosthetic stems and total joint replacements. Stainless steel (316L) and titanium-based alloys are among the materials employed in this category [4].

##### Total Joint Replacement

Total joint replacement involves using materials specifically selected with enhanced mechanical properties, such as creep strength or resistance to continuous deformation under sustained loading. This relates to the “Measurement of a materials’ ability to withstand sustained loading without significant continuous deformation” [28]. These materials also aim to minimize deterioration caused by corrosion and wear. In this context, the primary aim is to create a biomechanical environment that reduces disruption to the homeostatic balance in the bone and surrounding tissues. Biocompatible material requirements for this application can be extended to include how rapid the surrounding bone’s acceptance rate is for the replacement and the surrounding tissue’s prompt response to corrosion and wear debris of the replacement. Titanium and cobalt-chromium-based alloys have emerged as nearly ideal combinations of mechanical characteristics and metallic components for total joint replacement [2].

In total hip replacements, cement is used to secure the implantation components. However, due to a modulus mismatch, loosening can occur at the interface between the cement and bone. To address this, PMMA fixation allows patients to bear weight immediately after surgery. Surface properties, mechanical behavior, and osteocompatibility are all integral aspects of biocompatibility that require thorough investigation to develop novel bone biomaterials [29].

##### Spinal Implants

Spinal surgery has a long history, dating back to Jules Gerin’s initial efforts in repairing scoliosis in 1839. Our understanding of the spine has improved, altering surgical techniques and instruments. Spinal implants must be biostable and biocompatible. The materials for these implants are chosen based on stiffness and brittleness. Other important biomechanical factors include stiffness, fatigue, and the strain ratio. Common spinal implant materials are stainless steel, titanium, cobalt-chrome, nitinol, tantalum, and polyether-ketone. These materials are found to meet the requirements for spinal implants and especially the biocompatibility requirements. Table 3 highlights the differences and features of spinal implants [29].

The current generation of implants is typically constructed using a combination of cobalt-chromium molybdenum and ultrahigh molecular weight polyethylene to provide the necessary strength and durability. Additionally, a rough titanium surface coating is applied to stimulate bone formation, promoting the integration of the implant with the surrounding bone tissue. This alloy coating is a crucial element in ensuring the implant’s long-term success, which also falls into the concept of biocompatibility [30].

#### 3.1.4. Dentistry and Prosthetic Implants

The oral cavity, which serves as the site of long-term implantation and restoration procedures, poses unique challenges in terms of biocompatibility due to specific characteristics and processes occurring within it. These include the constant exposure of teeth to substances like saliva, bacteria, and food, which significantly influence the requirements for biocompatibility. Additionally, the oral cavity’s continuous exposure also leads to tissue instability and variations in temperature, pH levels, and other environmental aspects [31].

Biomaterials must meet several parameters beyond the basic physical and chemical standards to be deemed biocompatible for dental usage. They need to demonstrate durability and viability in aquatic settings. Moreover, while selecting dental filling materials, it is essential to consider the expected and potential adverse effects associated with their use [31]. However, adverse reactions can also affect dental personnel who handle certain materials, such as rubber products. After years of exposure to methacrylate-based materials, dental professionals have reported issues like dry, peeling, or cracking skin and generalized neuropathy [32].

##### Dental Implants

Dental implant materials must exhibit exceptional mechanical durability to endure the substantial stresses to which teeth are regularly subjected. Teeth experience the highest compressive stress within the body due to significant pressures concentrated on a small surface area. Therefore, the selected materials must have the capacity to withstand constant high-value compressive forces and additional forces during activities like shear and torque [33].

Historically, dental implants were categorized into two main types based on location and function: subperiosteal and endosseous tooth implants [33]. For these implants to be long-lasting and stable, they must establish a suitable connection with the surrounding tissues through osseointegration [34]. Osseointegration refers to the direct anatomical and functional integration between living bone and the surface of the load-bearing implant. It ensures implant stability and long-term therapeutic success. The process begins with the interlocking of the alveolar bone with the implant body and progresses through ongoing bone apposition and transformation towards the implant, ultimately leading to a biological attachment. This complex procedure profoundly influences bone development and preservation at the implant surface [35].

##### Endosseous Tooth Implants

Long-term dental implants can replace missing teeth when the natural tooth root is not viable. These implants are made from biomaterials introduced into the jawbone, creating a junction site between the material and the surrounding environment [34]. The ideal choice for a tooth replacement is a dental implant that closely mimics a natural tooth, although alternative options, such as dentures or false teeth, often lack stability and aesthetic appeal, making them a partial solution for patients [33].

An endosseous implant is a dental implant that is anchored in the jawbone. It is implanted into the jawbone and allowed to heal before an artificial tooth or crown is attached. This type of implant, known as an endosteal implant, closely resembles a natural tooth root [36]. Endosseous implants come in various designs, like self-tapping screws, a spiral screw-vent, and a blade-vent, to ensure immediate stabilization and enduring fixation. After approximately 14 months of rigid fixation, an appropriate crown is attached. Some implant systems involve burying the implant root in the extraction site, installing a post through a punctured hole in the gum tissue, and then creating the crown. However, despite the complex design, the success rate of this system is not higher than for other implants, such as blade-vents. Dental implants remain a popular choice [33].

Titanium and zirconia are two common materials used in dental implants. Titanium is a biocompatible metal known for its strength, light weight, and corrosion resistance. Zirconia is a biocompatible ceramic that is a good match for natural teeth color. Both materials are well tolerated by the body and can integrate with the surrounding bone tissue through osseointegration, which is essential for implant stability and long-term effectiveness. However, certain limitations exist with pure titanium implants, especially for small diameter and single-tooth implants, as they may be prone to fatigue fractures. To overcome these challenges, modifications have been made to these materials to meet the required characteristics for dental implants. For instance, the investigation of binary titanium zirconium alloys has shown promise in addressing the issues associated with small diameter implants [37].

##### Subperiosteal and Staple/Transosteal Implants

The second type of long-term dental implant is known as the “subperiosteal” implant. This name indicates that the foundation or frame of the implant is positioned beneath the gum line [38]. These implants addressed weak support in certain patients, aiming to provide enhanced support for dentures or other types of bridge treatments placed on top of these implants [33].

Titanium alloys are considered the gold standard for dental implant materials due to their excellent mechanical properties and high biocompatibility with the surrounding environment. However, there are cases where patients require additional support for implants or bridges, particularly in severe maxillary atrophy. Maxillary atrophy is significant bone resorption, sometimes accompanied by maxillary sinus expansion, resulting in inadequate ridge height, width, or both [39]. This poses challenges for conventional implants without needing bone graft surgery and alveolar reconstruction. In such cases, subperiosteal implants offer a viable alternative independent of the maxillary bone [40].

Metals like stainless steel, Co-Cr alloy, and Ti alloy are commonly used for subperiosteal implants due to their ease of manufacturing in standard dental laboratories [33].

##### Dental Restoration

Biocompatibility principles are also applied in dental restoration, which involves repairing the teeth affected by decay or cavities [31]. The materials used in dental restoration are known as restorative materials. Most of these materials are not directly set in contact with the surrounding tissues, except for certain materials like dentin and enamel [41].

##### Amalgam

Amalgam and composite materials are widely used in dental repairs. Amalgam fillings, composed of liquid mercury, silver, and other metals like copper and zinc, have been utilized for many years due to their affordability, durability, and ease of placement [41]. However, concerns have been raised regarding the potential toxicity of amalgam fillings, since they contain mercury as about half of their components, which is responsible for their silver appearance. Mercury vapor, known for its high volatility, can be released in small amounts from hardened fillings due to stress and tension during activities like eating and brushing [31]. Amalgam restorations have the potential to cause delayed hypersensitivity reactions, and regular exposure to mercury in these restorations may increase the risk of oral lichenoid diseases. Dental professionals working with amalgam are at risk of exposure to inorganic mercury, leading to higher urinary mercury levels and suspected signs of mercury poisoning. However, there is no significant association between urine mercury levels and self-reported memory problems. Studies have shown that occupational exposure to mercury vapor in dental offices does not damage white blood cells genetically [42].

##### Resin-Based Composites

Resin-based composites (RBCs) are a relatively recent development in restorative dentistry. These materials effectively fill cavities, especially for front teeth. They closely match the original tooth color, resulting in a pleasing appearance. RBCs comprise a combination of ceramics and polymers, with Bisphenol A (BPA) used as a component synthesizer. Using BPA and other potentially hazardous components as monomers has raised concerns regarding RBCs. However, extensive research has been conducted to investigate the harmful effects of these materials. BPA and other toxic materials were less harmful when placed in dentin. Dentin tubules are small hollow tubes or canals that allow heat, cold, and various foods to trigger the nerves and cells inside the tooth, leading to sensitivity when the protective enamel coating wears away [42]. Ongoing research is being conducted to explore this topic further [31].

#### 3.1.5. Biocompatible Alloys

An alloy is a substance formed by combining two or more elements, often metals, either in the form of a compound or a mixture. It is important to note that, in the case of steel, which is an alloy, carbon, a nonmetal, plays a significant role. These materials are engineered to become what is known as a biocompatible alloy [43]. Biocompatible alloys are carefully designed to coexist harmoniously within the human body, ensuring they do not provoke adverse reactions or toxic responses upon introduction. These substances must exhibit excellent corrosion resistance to withstand challenging physiological conditions, preventing any tendency for deterioration over extended periods. Additionally, they must possess the necessary mechanical strength to withstand physiological loads and pressures, avoiding the risk of fracture or distortion. A crucial requirement for biocompatible alloys is their ability to promote the integration of the implant with the adjacent bone tissue, facilitating proper recovery and ensuring long-term structural stability [44]. Biocompatible alloys find a wide range of applications in the field of biomedicine, including orthopedics, dental implants, cardiovascular devices, and surgical instruments. Titanium and its alloys are among the most well-known materials used in the orthopedic and orthodontic fields. This is primarily due to their high biocompatibility, good corrosion resistance, and excellent mechanical properties, including low density and low Young’s modulus. Titanium also demonstrates bioactive behavior, significantly enhancing the quality and longevity of implant use. This behavior is attributed to the gradual formation of a titanium hydrated oxide layer on the implant’s surface, facilitating the incorporation of calcium and phosphorus [45]. The new trends in alloys for biomedical applications include 3D printing techniques or additive manufacturing where, for example, powder bed fusion (PBF) is used to process enabled beta-titanium (β-Ti) alloys that have an increasing interest to tackle what is known as “stress shielding”, a phenomenon caused by a mismatch in a modulus between the implanted and the natural bones. The β-Ti alloys are promising due to their mechanical strength (lower elastic modulus) [46].

### 3.2. Short-Term Implants

Short-term implants are temporary, such as drug delivery systems, tissue contact parts, and orthopedic implants.

#### 3.2.1. Biodegradable Implanted Systems

Biodegradable implants are a type of material used in various devices that deteriorate. While typical devices prioritize stability, these systems can fail and be purged from the body. Therefore, selecting suitable materials for biodegradable implants is crucial to ensure they fulfill their function without causing harm [2].

Suture materials play a vital role in wound repair by providing support to healing tissues. However, there is no perfect suture material. Various factors need to be considered when choosing sutures, including tensile strength, tissue absorption, diameter, knot strength, security, coefficient of friction, plasticity and elasticity, handling, memory, tissue reactivity, capillarity, fluid absorption, and ease of removal. Sutures can be classified as absorbable or nonabsorbable. Commercially available absorbable sutures include polyglycolic acid, gut, polydioxanone, poliglecaprone, polyglycolide-trimethylene carbonate, polyglactin 910, and caprosyn. Nonabsorbable sutures include materials such as silk, braided polyester, polypropylene, nylon, stainless steel, and polybutester. There are also absorbable and nonabsorbable barbed sutures available [47].

One traditional example of a suture material is catgut, a protein fiber derived from the small intestines of animals such as sheep or oxen, which has long been used in surgical procedures. Despite its significant disadvantages, such as poor repeatability and aggressive tissue reaction, catgut was the sole recognized material for these types of devices for many years [13]. One of the most significant concerns with catgut is that it stiffens after drying, making it difficult to deal with. That it is derived from animals has raised ethical and health concerns [2].

Despite these drawbacks, recent developments have shown promising applications of catgut in implanted neurological devices and systems, particularly in sutures. Neurosurgeons have discovered that cat sutures, though initially challenging to work with, can be modified to possess characteristics that aid the surgical process. This has led to the increased use of catgut sutures in neurosurgery, potentially improving patient outcomes and reducing recovery times. Ongoing research aims to further explore the properties of catgut and develop new methods for their use, potentially finding applications in other medical fields. While catgut has limitations, its unique properties and potential benefits make it an area of focus for research and development in the medical field [12].

Biodegradable implant materials can undergo spontaneous disintegration, absorption, digestion, or expulsion within the human body, eliminating the need for subsequent implant removal surgeries once the surgical site has healed. However, these materials may have limitations if not modified. Many biodegradable materials, often polymers, lack the mechanical strength required to withstand the weight and pressure of the body, making them unsuitable for load-bearing applications. The choice of material is crucial in the development of these systems. For example, magnesium alloys have been explored as an alternative to temporary metallic orthopedic implants due to their acceptable mechanical properties. Magnesium alloys exhibit compatibility with human bone, providing comparable load-bearing capacity and stress distribution. However, their susceptibility to corrosion poses challenges that need to be addressed for their future successful use [12,48].

#### 3.2.2. Drug Delivery Systems

In the category of short-term implants, drug delivery systems play a significant role. It is essential to consider the influence of medications on the biocompatibility of these systems, especially when formulations involve a stationary depot. This is particularly relevant for long-acting local anesthetics. Various approaches have been employed to achieve the continuous release of medications like bupivacaine, including the use of polymeric particles, spray-dried lipid-protein-sugar particles, liposomes, cross-linked hyaluronic acid gels, and polysaccharide rheological blends. These delivery strategies typically result in minimal or no tissue damage and varying degrees of inflammation when unloaded. However, when loaded with bupivacaine, these systems might cause muscle injury to different extents. Therefore, in developing drug delivery systems, a thorough study of the medication and delivery method and their interaction is necessary to ensure optimal biocompatibility and minimize the risk of unwanted effects. Extensive testing and evaluation through preclinical and clinical trials are crucial to determine the safety and efficacy of these systems before their widespread use [49].

Given the direct interaction of these drug delivery systems with the patient’s body, achieving biocompatibility becomes a critical aim to investigate and enhance. Several examples of chemical and pharmaceutical materials have been used to develop biocompatibility in drug delivery systems. One approach involves modulating the surrounding tissue reactions using anti-inflammatory compounds, which can help reduce inflammation in and around the devices. However, efforts to produce more biocompatible materials have been hindered by a lack of understanding of the complex interactions between materials and tissues. Biocompatibility is not simply a matter of isolated interactions but encompasses various aspects, particularly in drug delivery systems, such as chemical product degradation and interactions with cells. Further research is needed to unravel these material–tissue interactions and determine the most effective strategies for achieving biocompatibility in drug delivery systems [49].

#### 3.2.3. Temporary Orthopedic Implants

Temporary orthopedic implants are commonly used when a patient’s bones are damaged during healing. These implants, including plates, screws, pins, cables, and intramedullary nails, serve a temporary purpose and are only utilized until the bone has healed [50].

Bone is a dynamic tissue capable of regenerating and restoring its biological and mechanical properties after injury. However, certain diseases, disorders, and traumas can cause damage to the skeletal system, leading to fractures and defects that increase the risk of mortality. In some cases, the presence or need for implants can also result in fractures or defects. Therefore, it is crucial to carefully design orthopedic devices to effectively treat skeletal trauma without causing harm to the patient [51].

Temporary orthopedic implants, also known as internal fixations, are relatively straightforward in their components, typically comprising plates of various sizes with holes. These holes are intended for placing screws and pins, which secure the plates to the bone to facilitate proper healing. Using screws and pins as fixations is necessary to withstand significant load forces and other types of forces [50]. It should be noted that there are different types of internal fixations for temporary orthopedic implants, depending on the location of the fracture or where they are used. For example, internal fixation may involve open reduction with plates and screws in case of a femoral fracture. These implants must be designed with considerations for biocompatibility, mechanical and surface qualities, and chemical and fracture properties. This ensures that the implant closely mimics the biomechanical characteristics of the bone and maintains its integrity for an extended period while integrating with the surrounding tissue as long as needed [51].

Given the skeletal system’s inherent capabilities, internal fracture repair biomaterials must withstand recurring stress. Metals, polymers, and ceramics have all been employed as orthopedic biomaterials, but metals are preferred because of their mechanical properties that provide essential stability. Specifically, titanium alloys, cobalt-chrome alloys, and chromium steel are the most commonly used metals, with titanium alloys and electropolished chromium steel being the preferred choices for fracture repair materials. Cobalt-chromium alloys are less used because of their complexity and high manufacturing costs [51].

The primary purpose of these implants is to aid the bone in its healing process, restoring the structural integrity and normal functionality of the injured tissues. Therefore, several factors must be considered during the production of these implant components, including corrosion resistance, wear resistance, mechanical properties, and osseointegration. The most critical factor is the biocompatibility of the material used. Table 4 outlines the main points and motivations for the implant design [50].

### 3.3. Tissue Engineering: Advancing Biocompatibility in Regenerative Medicine

Tissue engineering is a rapidly evolving field that combines scaffolds, cells, and physiologically active materials to create functional tissues. The main objective of tissue engineering is to build structures that can heal, sustain, or rejuvenate damaged tissues or organs [30]. As a more practical definition, “Tissue engineering is the creation of new tissue for the therapeutic reconstruction of the human body, by the deliberate and controlled stimulation of selected target cells through a systematic combination of molecular and mechanical signals” [2].

Considering the fundamental principles of tissue engineering, biocompatibility plays a crucial role. Unlike other fields that focus on stability or specific physical and mechanical functions, tissue engineering requires materials that can activate targeted cellular responses and initiate a cascade of reactions [2]. Therefore, the selection criteria for materials in tissue engineering are contingent upon understanding the target tissue’s natural environment and the material’s biomimetic properties. One essential component of tissue engineering is the use of scaffolds, which are synthetic three-dimensional (3D) structures made from polymeric materials. These scaffolds provide a multifunctional environment, mimicking the native tissue’s properties, cell signaling, and adhesion [52,53]. Electroactive biomaterials, such as polypyrrole, polyaniline, and other polymers, are employed in constructing these scaffolds, mimicking the extracellular matrix (ECM) of muscle cells [52].

Biomaterials used in tissue engineering can be classified into three categories: natural materials, synthetic materials, and hybrid materials, which combine natural and synthetic components. These materials undergo extensive processing and modification to impart functional properties and create porous scaffolds suitable for tissue engineering applications [54]. Resorbable polymers are the primary substrate materials in tissue engineering, while ceramics and metals have limited uses due to their persistence and poor formability. Commonly used polymers include natural protein and polysaccharide gels, resorbable synthetics, cross-linked hydrogels, and fibrous webs. Ceramics may apply to polymer substrates to enhance osteoconductivity. Various fabrication techniques, including traditional methods and rapid prototyping, are employed to create these scaffolds. Custom implants can sometimes be designed using radiographic images of the patient’s anatomy [55].

Synthetic tissues should be constructed with cells or components from the same species and tested in the target species. While this approach significantly reduces the risk of immunological reactions, it does not eliminate them entirely. For example, Harriger et al. utilized glutaraldehyde-cross-linked bovine collagen as a scaffold to seed human keratinocytes and fibroblasts, which were subsequently transplanted into athymic mice with full-thickness wounds [54]. Tissue engineering includes in vitro cell production and extracorporeal devices. The overarching goal is to achieve tissue and organ regeneration through innovative approaches [33].

## 4. Biocompatibility Testing: Assessing Compatibility and Ensuring the Safety of Hosts

The evaluation of biocompatibility is a complex process, as the compatibility of a material can vary depending on specific conditions, making it a gray area that necessitates rigorous testing [3]. Biocompatibility testing provides crucial insights into the interaction between materials and the biological system and the potential risks associated with their use [55]. Various parameters are considered when assessing a material for biocompatibility, which depend on its intended clinical application. For medical devices such as braces or prosthetic limbs, the material must be biocompatible and bifunctional, capable of performing multiple functions. Stability over time, the absence of degradation into harmful compounds, surface texture, crystallinity, moisture absorption, chemical properties, collapse resistance, surface charges, and stiffness are critical factors influencing a material’s compatibility with human tissue. Factors such as the administration method, location, and contact with specific cells or tissues also influence its potential for harm. Different criteria are evaluated when assessing materials for biocompatibility based on their major clinical application. For orthopedic use, properties such as texture, crystallinity, wettability, surface chemistry, breakdown products, charges, and stiffness must be considered. Interaction with the biological milieu of the target tissues, including protein adsorption, inflammatory processes, and contact with blood, as well as the duration and type of application, are also considered [4].

### 4.1. In Vivo vs. In Vitro Testing: Unveiling Material Safety

Biocompatibility testing is classified based on the environment in which it is conducted: in vitro or in vivo [55]. “In vivo” and “in vitro” tests refer to the location of the test. “In vivo” is an Italian term meaning “within living organisms” and pertains to tests performed on live organisms. Conversely, ”in vitro” testing refers to experiments conducted in a laboratory setting without the direct involvement of living organisms [56].

In vitro testing is crucial for assessing the safety of a product or service before its application in humans. Cellular and molecular tests are conducted to determine the safety of the product or service. This testing is conducted first to reduce the potential risks before any testing involving humans or organisms. By evaluating the cellular and molecular responses, the suitability of the product or service can be assessed [57].

In vivo testing examines the effects of a substance on a living animal, while in vitro testing looks at cells or tissues outside a living organism. The term “in vivo”, translated from Latin as “inside the living”, emphasizes that these experiments are conducted inside an animal, whether it be an animal model or a human volunteer [56]. Biomaterials must be evaluated beyond in vitro or in vivo testing. Unfortunately, in vitro testing does not eliminate the necessity for in vivo testing [56]. Both approaches are essential to comprehensively understand the performance and safety of biomaterials.

### 4.2. Tests of Various Material Properties: Evaluating the Biocompatibility

Biocompatibility testing is classified based on whether the tests are conducted outside or within the body, focusing on three critical areas: mechanical, chemical, and biological properties. As previously mentioned, each of these groups plays a crucial role in determining a material’s biocompatibility and suitability for its intended application. Tests may differ in required outcomes, aspects assessed, and conditions. Factors such as the country where the testing is conducted, the applicable standards, and the intended location of the device influence the specific tests that need to be performed.

### 4.3. Mechanical Properties Assessment: Ensuring Performance and Durability

Several factors must be considered when evaluating a material’s mechanical compatibility to ensure the optimal product performance and long-term durability. Critical considerations include tensile strength, hardness, static and fatigue resistance, and the material’s ability to withstand specific loads and pressures without failure. These mechanical characteristics directly impact the capabilities and functionality of a device or implant under various conditions. Thus, a rigorous evaluation of a material’s mechanical properties is crucial to achieving the required performance and longevity of the final product [58].

Mechanical testing plays a vital role in designing and evaluating medical devices that interact with biological tissues and biomaterials. It is essential to examine the mechanical properties of the biomaterials used in these devices throughout the design process. The persistence of these properties after implantation indicates biocompatibility, while any changes can provide valuable insights into the degradation process of biodegradable materials. Moreover, assessing the mechanical properties of host tissues can evaluate device safety and efficacy after in vivo implantation. Therefore, mechanical testing is essential to ensure the quality and safety of medical devices, including biological tissues and biomaterials [59]. These tests provide valuable insights into materials’ mechanical behavior and compatibility, ensuring their suitability for specific biomedical applications (Table 5).

#### 4.3.1. Chemical Testing

Chemical characterization testing studies extractable and leachable compounds from medical devices or materials. Extractable compounds are liberated when test materials are exposed to extraction solvents or more aggressive conditions than those encountered during clinical usage. On the other hand, leachable materials are discharged during actual clinical usage. It is essential to assess the medical equipment, component, or material biologically or chemically to understand its chemical composition and the potential migration or leaching of its elements and additives into patients’ bodily fluids or tissues [55].

In chemical analysis, two primary forms of characterization tests are direct material characterization and analytical methodologies designed to detect potentially emitted compounds from various devices. The initial battery of examinations focuses on evaluating the internal chemical properties of the materials. The latter procedure, known as extractable and leachable analysis (E&L), assesses the potential of compounds to seep out from a device. This analysis is further supported by a toxicological risk assessment that utilizes permissible exposure limits [61]. To ensure the comprehensive chemical characterization of materials, it is recommended to follow the standards provided by ISO 10993-18 and 17. These international standards offer a framework for evaluating the chemical composition of medical devices and their potential to release leachable chemical substances and impurities that may pose health risks to patients. The use of analytical methods, which are a collection of tests that aid in understanding the chemical characterization of materials, is advised [62].

##### General Steps and Uses

The suggested process for the chemical characterization of a device material involves a thorough analysis of the qualitative composition of each ingredient or material and an estimation of potential patient exposure. This requires conducting scientific research in a laboratory environment to determine the quantities of extractable potentially harmful components. Following these investigations, compiling a comprehensive material data file is crucial. The data obtained can ensure consistency in future production batches and reduce the need for traditional biological testing. The analytical characterization data can assess the overall biosafety of a medical apparatus, quantify the amounts of the substances that may be released into the device, assess conformance, determine material–device compatibility, and investigate the suitability of potential new materials for a proposed clinical purpose [62].

##### Tests

Characterization and analytical procedures are widely employed in various disciplines to identify and isolate substances or materials and explain their physical and chemical characteristics. These strategies aid in characterizing the qualities of materials, including whether they are crystalline [63]. Based on the major characteristics they address, these tests can be classified into three categories: extractable materials, bulk materials, and surface properties. The table below presents a set of generic categories for analytical tests and provides examples of specific tests performed within each category (Table 6).

#### 4.3.2. Biological Testing or Assessment

A biocompatibility assessment relies heavily on biological testing, which constitutes a crucial phase. The ISO biocompatibility evaluation matrix categorizes medical devices based on the duration and nature of their contact with the human body. It also includes a list of potential biological responses that must be examined and managed in their regulatory application for each device category [63].

Biological testing primarily aims to protect individuals from potential risks associated with using materials and medical devices, particularly implants. It is necessary to investigate these materials’ biological, local, and systemic effects and assess the devices’ biological safety. A comprehensive biological safety assessment must consider the type and duration of body contact. Manufacturers refer to the ISO 10993 series, which outlines the recommended approach for a biological evaluation, the endpoints that should be addressed, and more [61].

Professionals with substantial knowledge and expertise establish and document the strategy and content of biological assessment governance for medical devices. In line with the risk management strategy, criteria are developed to assess the suitability of materials for their intended use, and the adequacy of the material characterization is reviewed. A justification for the selection and/or exemption of tests is provided. The assessors determine the significance of the existing information and laboratory testing results, identify any additional information required to construct a comprehensive biological evaluation, and provide comprehensive conclusions regarding the biological safety of the medical device. Due to the diversity of medical devices, it is not always feasible for each device to undergo all tests within a specific category. Therefore, it is crucial to evaluate the specific characteristics of each device before conducting any testing [64].

##### Cytotoxicity

Cytotoxicity tests are often performed in vitro on isolated cells. The primary purpose of these tests is to assess the material’s potential to cause cell death or damage [65,66]. Cytotoxicity is recommended as a pilot project test and an essential indicator for the toxicity evaluation of medical devices due to its simplicity, rapidity, high sensitivity, and ability to spare animals from toxicity [65]. These tests are typically qualitative, with the most commonly recommended tests focusing on the density of the test material.

Recent research on cytotoxicity has shown that quantitative evaluations yield better outcomes than qualitative evaluations. Therefore, the colorimetric assay, commonly known as the MTT assay, has been identified as the preferred approach. However, the MTT test has limitations, such as its inability to detect cellular damage in its early stages and its reliance on detecting cell death only [65].

##### Sensitization Assays

Sensitization assays are a group of tests used to detect whether a substance contains compounds that may produce undesirable effects after repeated or prolonged exposure. These tests involve immunological systems and can be conducted using specific compounds from the test material, the test material itself, or test material extracts. Similar to cytotoxicity assays, these tests have different variations, depending on the type of contact the material is expected to have [65].

##### Irritation Tests

Tests measure how irritating items are on animal skin or mucous membranes. The mode of device exposure (through the skin, eye, or mucosa) and the duration of contact should align with the expected clinical use, although it is sometimes recommended to overestimate the exposure conditions to provide some level of preventive protection for patients. It should be noted that all experiments are conducted on animals as part of preclinical research. The scoring system used may vary depending on the procedure [65].

##### Subchronic Toxicity Tests

Subchronic toxicity tests are employed to identify potential negative impacts resulting from prolonged or multiple exposures to test materials and/or extracts, covering a duration of up to 10% of the complete life cycle of the experimental animal, for example. Experimental research conducted on rats has suggested a maximum duration of 90 days. When selecting an animal model for subchronic toxicity assessment, it is crucial to consider the realistic application scenarios of a medical device. Determining suitable animal models depends on specific circumstances and requires an individualized evaluation. All permanent devices require subchronic testing, and additional consideration may be warranted for devices that exhibit prolonged integration with internal tissues [65].

Various protocols are available for these types of tests. For example, a specific laboratory specialized in biocompatibility testing offers two standard protocols that differ based on the method of administration. One protocol utilizes the intraperitoneal administration method [58], which involves the injection of a pharmacological drug into the peritoneal cavity and is commonly used in rat research due to its faster absorption rate. This approach is easy to learn and minimally stressful for the animals. The rodent is restrained in a supine position, with its head positioned lower than the rest of its body. The needle is then inserted at a 10 degree angle into the lower abdominal region [67,68]. The second protocol employs the intravenous route of administration, commonly known as tail vein injections [68].

##### Genotoxicity

Genotoxicity assessments involve a set of in vitro and in vivo studies aimed at identifying mutagens and materials that may directly or indirectly cause genetic harm through various mechanisms. Such damage can affect somatic or germline cells, increasing the cancer risk or causing inheritable abnormalities. The mutagenicity of a substance is closely linked to its carcinogenicity, which will be discussed in later sections. Genotoxic effects can be classified as point mutations along DNA strands, DNA structural damage, or chromosomal structure damage. Several tests have been developed to evaluate whether such damage has occurred, and these tests are conducted as a battery of assays. When selecting a battery of genotoxicity tests, examining the regulatory requirements of the specific agency to which the report will be submitted is crucial. Due to the cost of such testing, it is strongly advised to consult with the FDA reviewer before conducting any testing [65,66].

The table below presents a collection of genotoxicity tests that can be performed, along with their differences based on ISO 10933-1, which provides recommended standards for assessing potential genotoxicity for specific devices or materials. It should be noted that, according to these parameters, one test may be sufficient sometimes while multiple tests may be required in others, depending on the duration of contact and the criticality of the direct contact environment (Table 7).

##### Implantation Tests

Implantation tests evaluate the potential localized pathological effects on live tissue caused by a biomaterial or medical device sample when implanted or surgically placed at a suitable site or tissue for its intended application [69]. These tests assess the safety of medical devices or materials in contact with living tissue for medical purposes, excluding the skin. Surgical tools such as stitches, clamps, and devices inserted into the body during surgeries are tested. Implant surgeries assess the performance of materials that dissolve and those that do not. A histopathological analysis is used in these tests [65].

##### Hemocompatibility

The evaluation of hemocompatibility is crucial, because harmful materials can adversely affect various cell types found in the blood. Hemolysis can decrease the oxygen transport capacity through mechanical impairment or material-related processes. Adverse responses involving white blood cells can hinder pathogen clearance. Solubilized proteins in the blood play essential roles, such as activating the complement system for pathogen clearance, initiating inflammation, and initiating the clotting cascade to maintain tissue repair and limit fluid loss. Impeding these biological systems can negatively impact the physiological functioning of the organism. ISO 10993-4 specifies specific examinations based on the blood contact group of the device to achieve hemocompatibility. Thrombosis, coagulation, platelets, hematology, and immunology tests are recommended to be performed for any form of interaction [70].

It is important to recognize that all materials are incompatible with blood, as they can cause hemolysis by disrupting blood cells; activating coagulation pathways, resulting in thrombogenicity; or triggering the complement system. The table below shows some tests that can be performed to assess the hemocompatibility of a material and its differences. It should be noted that these tests sometimes do not meet ISO standards, so additional blood compatibility tests and in vivo studies may be required (Table 8) [65].

Table 9 presents examples of implant devices and the test categories that must be performed to test materials that are incompatible with blood if they can cause hemolysis [65].

##### Carcinogenicity Tests

Carcinogenicity tests are used to assess the potential of experimental compounds and/or extracts from single or multiple exposures to cause oncogenic effects during the life cycle of the testing organism. Carcinogenicity testing for devices remains a controversial topic due to the inherent challenges and costs associated with the procedure. Manufacturers can provide an alternative to extensive testing for the carcinogenicity of their devices. Carcinogenic compounds can cause malignant tumors, increase the frequency or severity of tumor occurrence, or speed up the onset of tumor manifestation through various absorption routes, such as inhalation, ingestion, topical application, or injection [65,71].

Carcinogenesis involves a complex series of events that lead to the transformation of normal cells into malignant cancer cells. This process occurs over multiple stages and is characterized by intricate biological interactions influenced by factors such as genetics, age, dietary habits, environmental exposures, and hormonal imbalances. The induction of cancer involves genetic alterations resulting from direct or indirect sources. Carcinogens can be classified into two distinct groups based on their mode of action: genotoxic carcinogens and nongenotoxic carcinogens. Genotoxic carcinogens interact with DNA or the cellular apparatus, disrupting the integrity of the genome. Nongenotoxic carcinogens exert their effects through alternative mechanisms that do not involve direct DNA modifications [71].

##### Reproductive and Developmental Toxicity Tests

Reproductive and developmental toxicity tests assess the effects of medical devices, materials, and/or their extracts on reproductive function, embryonic development (teratogenicity), and fertility [65,66]. Prenatal and early postnatal development refer to the periods of growth and development that occur before and immediately after birth. Devices that come into constant contact with internal tissues often require specific examinations. If a device has the potential to impair the subject’s reproductive potential, reproductive/developmental toxicity tests or bioassays must be conducted comprehensively. It is recommended to perform such assessments for devices and drugs used throughout the gestational period. The device’s application site is the main criterion for testing. The ISO 10993-1 standard provides the procedures for assessing reproductive and developmental toxicity [66].

##### Biodegradation Tests

Biodegradation tests are considered essential in certain situations. These circumstances include (a) when the device contains a biodegradable component; (b) when the device is intended for implantation exceeding 30 days; or (c) when a comprehensive analysis of the material composition indicates the possibility of releasing toxic substances upon contact with the body—in such cases, explaining and documenting the various parameters that influence the degradation rate and identifying the contributing factors to biodegradation [66].

Replicating biodegradation mechanisms in vitro is recommended to determine degradation rates and the release of potentially harmful materials for the performance evaluation. In some cases, in vivo assessments may be necessary to evaluate the biodegradation of a material. The need for biodegradation tests may be obviated if the potential sources of degradation are present in expected quantities and their generation rate is similar to what has been demonstrated to be sustainable in previous clinical applications. ISO 10993-9 provides a widely used framework for conducting biodegradation assessments, while ISO 10993-13, ISO 10993-14, and ISO 10993-15 offer specific in vitro procedures for evaluating biodegradation in polymers, ceramics, and metals, respectively [66].

##### Toxicokinetic Studies or Chronic Toxicity Tests

Toxicokinetic studies employ physiologically based pharmacokinetic (PBPK) models to assess the absorption, distribution, metabolism, and excretion (ADME) of hazardous materials. These studies help define the concentrations of toxicants in the target organ and assess the associated hazards. When extrapolating test results to various characteristics such as gender, age, species, and doses/exposure, expert opinions are crucial. In vivo toxicokinetic studies combined with in vitro biodegradation data may be required to understand the absorption, distribution, metabolism, and elimination of leachable and degradation products from medical devices. ISO 10993-16 recommends this approach. It is advisable to evaluate the in vitro degradation before conducting toxicokinetic investigations [66].

Toxicokinetic evaluations are necessary for bioresorbable implants or devices that show degradation anomalies, leachable migration, or the release of hazardous compounds during use. The use of animal tissue in toxicokinetic studies is decreasing due to ethical and analytical reasons. If levels of safe clinical exposure can be achieved using a device or material, and there is appropriate toxicological or toxicokinetic data available or based on experience with the material or device, toxicokinetic studies may not be necessary. However, toxicokinetic studies may be required for biodegradable materials with increased amounts of released degradation products and leachables. The current research is focused on developing methods for detecting and quantifying degradation products and leachables in accordance with ISO 10993-16 [66].

## 5. Regulatory Affairs Organizations That Deal with Biocompatibility and Their Focus

Testing for biocompatibility is an essential part of the regulatory process for medical devices to ensure their safety and suitability for use by people. Several regulatory affairs organizations are responsible for setting biocompatibility testing criteria and guidelines. These organizations focus primarily on evaluating the potential biological risks associated with medical devices and materials.

### 5.1. ISO, FDA, and TÜV SÜD

The International Organization for Standardization (ISO), the United States Food and Drug Administration (FDA), and TÜV SÜD are prominent regulatory bodies involved in establishing biocompatibility testing standards. ISO is an independent, nongovernmental organization that develops and publishes international standards for various products and services, including medical devices. The ISO 10993 series is the most widely used standard for assessing the biocompatibility of medical devices. It provides a set of tests designed to evaluate the potential biological hazards associated with a medical device. ISO 10993 is recognized and utilized by regulatory authorities worldwide [63].

In the United States, the FDA is responsible for medical device regulation. Biocompatibility testing is crucial to the pre-market approval process for medical products. The FDA requires manufacturers to conduct biocompatibility testing to determine the potential adverse effects of a device on the human body. The FDA has published its own version of the ISO 10993 standard, which mandates biocompatibility testing in line with ISO 10993. The FDA also provides guidance on biocompatibility testing and assessment, including the use of nontraditional animal testing methodologies [66].

TÜV SÜD is a European regulatory authority providing medical device certification and testing services. TÜV SÜD has established its own biocompatibility testing standards based on the ISO 10993 series. Manufacturers can submit their products to TÜV SÜD for testing and certification to ensure compliance with European Union regulations. Although TÜV SÜD certification is not mandatory, it can be beneficial for companies seeking to market their products in the European Union. TÜV SÜD certification verifies that a device has undergone a thorough evaluation and meets the safety and efficacy standards of the European Union [72].

### 5.2. The Focus of Biocompatibility Evaluation

The common focus of regulatory affairs organizations like the FDA and ISO is to assess the biocompatibility of materials and devices in order to minimize potential risks and harm to patients. While they evaluate different attributes and endpoints, their objective remains the same: ensuring the safety and compatibility of medical devices with the human body.

#### 5.2.1. FDA and ISO 10993

Over the years, the FDA has issued a nonbinding document that comprehensively defines the set of globally accepted guidelines for evaluating the biocompatibility of medical devices and materials, known as “ISO 10993”. Divided into 33 parts, various standards focus on the biological assessment of these devices and materials, since their considerations are primarily for the potential biological consequences on the patient’s or user’s health [66].

The ISO 10993 standard series has standardized the biocompatibility testing of medical equipment. Part 1 of the standard includes a framework for biological assessment planning and guidelines for selecting suitable tests. The subsequent sections recommend ways for conducting biological testing. The ISO 10993-1 standard was amended in 2009 to prioritize the use of chemical component testing and in vitro models where they give similarly relevant information as in vivo models. This method analyzes preexisting information before deciding if biocompatibility testing is required. As scientific information about the underlying processes of tissue responses has increased, the FDA supports this modification [66].

Based on the FDA’s guidelines and ISO 10993 standards, medical devices are categorized according to three major factors: the type of device; the location of contact with the body (blood, tissues, or skin); and the duration of contact between the material or device and the patient. The evaluation of devices is often conducted using qualitative tests, with the recommended tests focusing on specific aspects related to the category and duration of contact. The following table compares the differences between these tests, which are typically performed by specialized laboratories conducting biocompatibility testing. Table 10 explains such categorization by the FDA and ISO-10993 [73].

Based on these criteria, the ISO and, later, the FDA proposed a framework for biological tests or assessments for biocompatibility that may be useful. It was discovered that, because of the differences in categories, different technologies or materials require distinct testing, and varied biological end materials cause precise assessments. Because it is simply a framework, some devices may require more testing. The following two tables, Table 11 and Table 12, present a sample of the FDA endpoints of evaluation performed for implant devices [73].

Besides these endpoints, the FDA and ISO recommend evaluating and addressing reproductive and developmental toxicity, particularly if the material has a history of reproductive or developmental toxicity, such as in pregnant women, and if the device or its components degrade in the body [66].

#### 5.2.2. TÜV SÜD

TÜV SÜD tests new medical products, equipment, and newly modified devices for biocompatibility before they are admitted into the global market. TÜV SÜD, like the FDA, provides a framework for evaluating biocompatibility for devices and materials that references the standards previously established by the ISO 10993 series. Furthermore, TÜV SÜD’s supplied services and framework are in accordance with ISO 17025 and GLP, which stands for Good Laboratory Practice and are regulatory standards establishing the minimum criteria for planning, performing, and reporting nonclinical safety investigations [74].

Manufacturers can begin selling confidently with TÜV SÜD results of a biological risk assessment for a device or material, because these results meet the biocompatibility testing requirements of the International Organization for Standardization (ISO), the United States Food and Drug Administration (FDA), and the American Society for Testing and Materials (ASTM). Table 13 below is an example of an assessment performed by TÜV SÜD to assess potential dangers, as well as the standards to which they adhere [67].

According to the different regulatory bodies, the abovementioned assessments ensure that, if the tested devices or materials meet the required biocompatibility standards, they have safe applications. This allows manufacturers to confidently market their products, knowing they have undergone a thorough biological risk assessment.

## 6. Comments, Points for Consideration, and What Is New in This Review

Developing safe and effective medical devices requires biocompatibility. Biocompatibility refers to the ability of a material to coexist with biological tissue without causing harm. The interaction between materials and biological tissues involves multiple mechanisms, including chemical, metabolic, physiological, and physical processes. The foreign body response (FBR) provides a framework for understanding how the body responds to foreign materials. To determine the influence of material properties on the body’s reaction, researchers focus on both bulk material properties and surface material characteristics. Biocompatibility is a complex concept that considers patient characteristics and the therapeutic properties of the material. It is an essential aspect of the design process, as it can affect device behavior and patient tolerance. Therefore, when designing medical devices, consideration should be given to both bulk material properties and surface material characteristics.

The available information suggests no explicit instructions on selecting the set of tests to determine biocompatibility and compliance with regulations. To address this issue, a flowchart offers a simple “yes or no” decision-making process for manufacturers. The flowchart below suggests a clear set of tests addressing the three major potential risk categories: mechanical, biological, and chemical. The flowchart (Appendix A) is based on the information presented in the previous sections and incorporates concepts from sources such as the Williams Dictionary of Biomaterials and regulatory affairs organizations like the FDA and ISO.

To ensure clarity regarding the flowchart, the following points should be noted:

1. The flowchart uses information from the FDA and ISO.
2. The flowchart simplifies the understanding of the recommended testing framework.
3. The flowchart lacks specific documents for the ISO 10933 series. It is based on an understanding of the concepts and preferred characteristics.
4. The flowchart specifically focuses on the tests required for implantable devices.

## 7. Conclusions

In conclusion, biocompatibility is a complex concept that refers to the ability of a material to function safely within the human body. It is important to understand material–tissue interactions to create safe and effective medical devices. Various factors, including patient characteristics and material properties, influence biocompatibility. Researchers study bulk and surface characteristics to understand the body’s reactions to medical devices. The five foreign body response (FBR) phases explain the interactions between materials and human tissues. Defining and implementing biocompatibility is challenging, yet necessary.

This review article presents a valuable contribution to the field of biomaterials by presenting a Python code (Appendix B) designed to help biomaterialists select the most appropriate biocompatibility test for implants. The code is a powerful tool that enables researchers and medical professionals to make relevant decisions. The code provides a comprehensive evaluation framework for assessing implant biocompatibility by considering factors such as the material properties, intended application, and regulatory requirements. An accompanying flowchart visually summarizes the decision-making process, enhancing the code’s accessibility and utility. A flowchart offers an efficient and user-friendly way to navigate implant biocompatibility testing. Biocompatibility evaluations lead to safer implants.

Future recommendations for biocompatibility in medical devices include advances in material characterization, the development of standardized test protocols, the long-term evaluation of biocompatibility, the integration of advanced technologies, and the resolution of individual changes. These insights will help researchers and industry professionals advance their understanding of biocompatibility and benefit patients’ health and well-being, contributing to the development of safer and more effective medical devices. Research is essential for material characterization, testing protocols, biocompatibility, technology integration, and variable-resolution.

## Figures and Tables

**Table 1:** The history of materials used in medical applications.

Application	Material	Who and When	Additional Notes
Sutures	Linen	Early Egyptian civilization [10].	Linen was discovered to be extremely complimentary to human cells. It was extremely absorbent and capable of reducing fever as it is antibacterial. In addition, it can keep dust from passing and other properties that let it be handy [11].
	Catgut	Used by Europeans during the Middle Ages [10].	Catgut’s capacity for withstanding tension and its ability to last make it the perfect material for sutures [12].
	Heads of large biting ants	Famously seen in South Africa and India [10].	Ants were used as “suturing devices”, because they had powerful mandibles. They were put extremely close to the wound and bit directly against both margins of the cut, reducing the distance between them. The ant’s body would split after a certain period, leaving the head and bite firmly in place to preserve a closed wound [13].
Hip prostheses	Wood	The first attempt at repairing a broken hip is made in 1840 [14].	A wooden block did not replace any biological tissue, but it was inserted between the broken ends of the hip! Following such a procedure, more and more foreign and biological elements were inserted [15].
	Ivory	Glück’s implant was a ball and socket prosthesis in 1880 [14].	Ivory is a substance found in various biological components, including elephant tusks, boar teeth, and many more. It is recognized to have various desirable features, including mechanical properties, machinability, and homogeneity [8].
	Glass	Smith-Petersen used to fit with the hip joint in 1925 [10].	Despite its moderate biocompatibility, glass was unsuccessful as a hip prosthesis material. The smooth surface of the 1925 glass as an entire hip prosthesis quickly cracked under the pressures exerted by the joint [16].
Dental Restorative materials	Gold	In 1795, Robert Wolfendale was the first to employ gold (gold foil) for tooth repair.Later, in 1855, gold foil was found to be a cohesive substanc [17].	Gold foil is composed of pure gold and is desirable to use in the tooth restoration process with cold work. This temperature range is beneficial, which results in an exact filling. However, gold mechanical resistance is incompatible with the application, so it was only employed for extremely tiny holes [18].
	Zirconia	In 1789, German chemists learned how to use it [19].	Approximately 20 years ago, zirconia emerged as a promising restorative dental material due to its superior mechanical qualities. The decision to employ zirconia was primarily driven by its exceptional strength, making it suitable for load-bearing applications. However, despite its advantageous mechanical properties, zirconia fell short in terms of aesthetic appearance due to its opaque coloration [19].

**Table 2:** The main components of a mechanical valve, summarized from [24].

Component	Description	Examples
Locking Element	One or more moving parts that facilitate the valve’s opening and closing.	- Ball - Single disk - Circular or semi-circular set of petals
Cover	The cage or ring that houses the locking element, allowing it to move.	- Graphite-made cage - Metal-made cage
Base	A ring-shaped component bordered with synthetic fabric, providing the foundation for the valve’s assembly.	- Ring with synthetic fabric edging

**Table 3:** Various types of spinal implants, summarized from [30].

Device	Purpose	Materials	Biomechanical Properties	Advantages	Disadvantages
Cage	Used as a stabilizer to distribute forces between vertebral bodies and to restore space between intervertebral and foramina space.	It is typically made from metal, ceramic, plastic, most commonly PEEK, titanium, and stainless steel.	Elastic modulus is similar to bone; radiolucent; good load-sharing; minimally invasive; preserves normal spinal anatomy.	They provide a graft for vertebrae to refuse and heal when the intervertebral disc has failed. Because of their porosity, they allow the bone to grow through them.	Some materials might be hydrophobic and unable to bond to bone for solid fusion.
Pedicle Screws	Provide rigid attachment between vertebrae and rod; allows for precise correction and alignment. Allow the redirection of forces.	Titanium, especially TiAl4V, stainless steel, cobalt-chromium.	High bending and torsional strength; low profile; rigid fixation; improved fusion rates; reduced rates of pseudarthrosis.	They can withstand significant forces and loads which are used in scoliosis.	There is a high possibility of loosening the screw, pulling out, or breaking, that might affect bone healing.
Spinal Rods	Adds stability to spinal implant structure; contoured to the patient’s spine.	Titanium, PEEK, stainless steel, cobalt-chromium, nitinol.	Biocompatible; improved biomechanical properties; minimal artifact on imaging; improved sagittal realignment.	The choice of material provides the patient with a wide range or customized characteristics.	Risk of fatigue, fractures, deformation; notch sensitivity; difficulty in identifying faults or breaks; risk of pseudarthrosis; the possibility of leaving weakness that affects overall durability.
Spinal Plates	Adds stability to spinal implant structure; screws into vertebral bodies to help restore normal alignment.	Titanium, stainless steel.	Rigid fixation; improved fusion rates.	—	—

**Table 4:** Key factors in the implant design and their significance in biocompatibility, summarized from [50].

The Key Properties	The Purpose	Actions Taken
Mechanical properties	Ensure endurance and functionality under operating conditions.	- Measure Young’s modulus, yield strength, ultimate tensile strength, fracture toughness, elongation at break, and fatigue resistance. - Engineer implant materials to have Young’s modulus equal to that of human bones for reduced bone resorption and implant loosening. - Strengthen fatigue strength to endure cyclic loading and prevent fatigue fracture, a main source of early implant failure. - Ensure good fracture toughness to prevent crack propagation under load and facilitate manufacturability.
Wear resistance	Minimize implant failure due to wear debris and osteolysis.	- Select materials with high yield strength and Young’s modulus for strong wear resistance and hardness. - Consider factors that reduce wear debris entering periprosthetic tissue and causing unfavorable biological responses. - Critical for joint replacements and fixation devices to minimize implant loosening and premature failure.
Corrosion resistance	Ensure implant longevity and prevent the release of harmful substances.	- Develop highly corrosion-resistant implant materials for physiological environments. - Use nontoxic alloying elements and minimize trace element release during the implant’s lifespan. - Consider materials with regulated degradation rates for temporary orthopedic implants.
Biocompatibility	Avoid toxicity and immune system-triggered complications.	- Select nontoxic alloys for implants to avoid immune responses. - Reduce inflammation caused by implant wear. - Prioritize corrosion and wear resistance to ensure good biocompatibility.
osseointegration	Facilitate integration with a neighboring bone for stability.	- Make a surface that works with bone tissue to stop osteolysis and maintain long-term stability.

**Table 5:** Examples of mechanical tests, summarized from [60].

Mechanical Test	Description	Additional Information
Tensile Test	Examining the stress, strain, and yield deformation of materials under tension. A sample is pulled until it breaks while measuring the applied force and deformation.	The test standards vary depending on the material, such as ASTM D638 / ISO 527-2 for reinforced plastics, ASTM D412 / ISO 37 for vulcanized and thermoplastic rubber, and ASTM E8 / ASTM A370/ISO 6892 for metals [60].
Compression Test	Determine compressive strength, stiffness, and deformation of materials. A sample is compressed until it breaks while measuring the applied force and deformation.	ASTM D3574 covers flexible cellular materials, ASTM D695-15 covers rigid plastics, AITM 0010 covers 2-Inch Concrete Cubes, and ISO 844 covers rigid cellular plastics [60].
Torsion Test	Measures the behavior of materials under torsional load (angular) to determine their torsional strength, stiffness, and ductility. The test provides information about shear modulus of elasticity, shear yield strength, shear strength, and more.	Various types of torsion tests are conducted, including torsion only, axial torsion, and failure tests, depending on the specific requirements of the material or device being tested.
Fatigue Test	Measures the behavior of materials under cyclic load applied at different angles to determine their fatigue strength and fatigue life. A sample is subjected to repeated loading and unloading cycles until it fails while measuring the applied stress and number of cycles.	The results of fatigue tests are typically presented in the form of a graph showing the number of cycles to failure plotted against the amplitude of the cyclic stress.
Fracture Test	Measures the energy required to cause an already cracked material to break fully. This test helps determine the material’s ability to resist fracturing and provides insights into brittle fracture behavior and grain size examination.	Fracture tests are conducted to assess the fracture toughness and brittleness of the material and to study the grain structure and any potential defects.
Hardness Test	Measures the ability of materials to resist indentation, scratching, or deformation. Different hardness tests, such as Brinell, Rockwell, and Vickers, employ different methods to measure hardness.	Hardness tests assess the material’s resistance to indentation or deformation, with specific test methods chosen based on the material and the desired hardness scale.
Impact Test	Measures the behavior of materials under sudden impact or shock load to determine their impact strength and toughness. A sample is subjected to a sudden impact or shock while measuring the energy absorbed by the sample.	There are two common impact tests: the Charpy and Izod tests. Both involve fracturing the material and measuring the energy absorbed during fracture to determine its impact resistance.
Creep test	Also known as a stress–relaxation test, it provides insights into the behavior of a material under constant stress.	Creep tests involve subjecting the material to constant stress or load for an extended period and measuring the resulting deformation or relaxation over time. Creep behavior is important for understanding long-term material performance.
Nondestructive testing	Nondestructive testing methods assess a material’s mechanical properties without damaging the original material.	Nondestructive testing techniques, such as acoustic emission testing, electromagnetic testing, and leak testing, are employed to evaluate the mechanical properties of materials without causing any permanent damage. These tests are valuable for quality control and inspection purposes.

**Table 6:** Chemical characteristics categories and associated tests, summarized from [62].

Category	Examples
Traditional Extractable Material Characterization	USP (United States Pharmacopeia) Physicochemical Test Panel for Elastomeric Closures for Injections USP Polyethylene Containers Tests–Heavy Metals and Nonvolatile Residues Indirect Food Additives and Polymers Extractables (21CFR Part 177 Sterilant Residues–Ethylene Oxide, Ethylene Chlorohydrin, Ethylene Glycol
Tests Procedures for Extractable Material	Liquid Chromatography Infrared Spectroscopy (IR) Mass Spectrometry Residual Solvents Atomic Absorption Spectroscopy (AAS) Inductively coupled Plasma Spectroscopy (ICP)
Bulk Material Characterization	Atomic Absorption Spectroscopy (AAS) Inductively coupled Plasma Spectroscopy (ICP) Thermal Analysis Infrared Spectroscopy Analysis to identify and estimate the Gross Composition (For example, Reflectance Spectroscopy, Transmission Spectroscopy
Surface Characterization	IR Reflectance Spectroscopy Scanning Electron Microscopy (SEM)

**Table 7:** Distinct types of genotoxicity tests, summarized from [65].

Type of Test	Description
Ames Test	Detects point mutations using Salmonella typhimurium bacterial strains sensitive to mutagens.
Mouse Lymphoma Assay	It uses mammalian cells to detect point mutations and can detect clastogenic lesions in genes.
HGPRT Assay	It uses mammalian cells to detect point mutations.
Unscheduled DNA Synthesis (UDS) Assay	Detects DNA damage and repair using both in vitro and in vivo methods.
Chromosomal Aberration Assay	Allows direct observation of chromosome damage using both in vitro and in vivo methods.
Mouse Micronucleus Assay	Detects chromosome damage using mammalian cells.

**Table 8:** Hemocompatibility tests, summarized from [65].

Test Name	Recommended for	Purpose
Hemolysis assay	All devices except those that do not have direct contact with blood cells	Measures the damage to red blood cells when exposed to materials or their extracts and compares it to positive and negative controls.
Coagulation assays	All devices with blood	Measures the effect of the test article on human blood coagulation time.
Prothrombin Time Assay	All devices with blood	General screening test for the detection of coagulation abnormalities in the extrinsic pathway.
Partial Thromboplastin Time Assay	All devices with blood	Detects coagulation abnormalities in the intrinsic pathway.
Thrombogenicity test	Devices unsuited to in vivo	Required tests in coagulation, platelets, hematology, and complement system categories. The most common test for thrombogenicity is the in vivo method.
Complement activation	Implant devices	In vitro assay to measure complement activations in the human plasma due to exposure of the plasma to the test article or an extract. Measures complement activation.

**Table 9:** Hemolysis testing, summarized from [65].

Device Examples	Test Category				
	Thrombosis	Coagulation	Platelets	Hematology	Complement System
Annuloplasty rings, mechanical heart valves	x			x ^a^	
Intra-aortic balloon pumps	x	x	x	x	x
Total artificial hearts, ventricular-assist devices	x			x	
Embolization devices				x ^a^	
Endovascular grafts	x			x ^a^	
Implantable defibrillators and cardioverters	x			x ^a^	
Pacemaker leads	x			x ^a^	
Leukocyte removal filter		x	x	x ^a^	
Prosthetic (synthetic) vascular grafts and patches, including arteriovenous shunts	x			x ^a^	

^a^—Hemolysis testing only.

**Table 10:** FDA and ISO medical device categorization, summarized from [73].

	Category	Contact Location	The Duration of Contact
Meaning	Nature of body contact	It refers to whether it directly has contact with the body in terms of blood, tissues, or skin.	Refers to the duration of contact between the material and the patient.
Categories	Surface device External Communicating device Implants	It is different based on the category.	Limited (less than 24 h) Prolonged (over 24 h but less than 30 days) Permanent (over 30 days)

**Table 11:** Framework of evaluation for implant devices that interact directly with tissue/bone, summarized from [73].

Biological Effect	Limited Duration	Prolonged Duration	Permanent
Cytotoxicity	✔	✔	✔
Sensitization	✔	✔	✔
Irritation or Intracutaneous Reactivity	✔	✔	✔
Acute Systemic Toxicity	✔	✔	✔
Material-Mediated Pyrogenicity	✔	✔	✔
Subacute/Subchronic Toxicity		✔	✔
Genotoxicity		✔	✔
Implantation		✔	✔
Chronic Toxicity			✔
Carcinogenicity			✔

**Table 12:** Framework of evaluation for implant devices that directly interact with blood, summarized from [73].

Biological Effect	Limited Duration	Prolonged Duration	Permanent
Cytotoxicity	✔	✔	✔
Sensitization	✔	✔	✔
Irritation or Intracutaneous Reactivity	✔	✔	✔
Acute Systemic Toxicity	✔	✔	✔
Material-Mediated Pyrogenicity	✔	✔	✔
Subacute/Subchronic Toxicity		✔	✔
Genotoxicity	✔	✔	✔
Implantation	✔	✔	✔
Hemocompatibility	✔	✔	✔
Chronic Toxicity			✔
Carcinogenicity			✔

**Table 13:** TÜV SÜD sample of a risk assessment and their standards, summarized from [67].

Test	Standards	What Does It Evaluate?
Cytotoxicity	ISO 10993-5 [67]	Test for toxicity of medical device or material on cell culture.
Genotoxicity	ISO 10993-3 and FDA [67]	Test for toxins that affect the genetic material of cells.
Hemocompatibility	ISO 10993-4 and ASTM [67]	Test for effects of blood-contacting medical devices on blood.
Irritation and Sensitization	ISO 10993-10 [67]	Test for skin irritability and adverse cutaneous reactions.
Systemic Effects	ISO 10993-11 and ASTM [67]	Test for effects of medical devices on the body, for example, the possibility of fever and toxicity.
Implantation	ISO 10993-6 [67]	Test for effects of medical devices on surrounding tissue at various levels of visibility.

**Figure A1:**
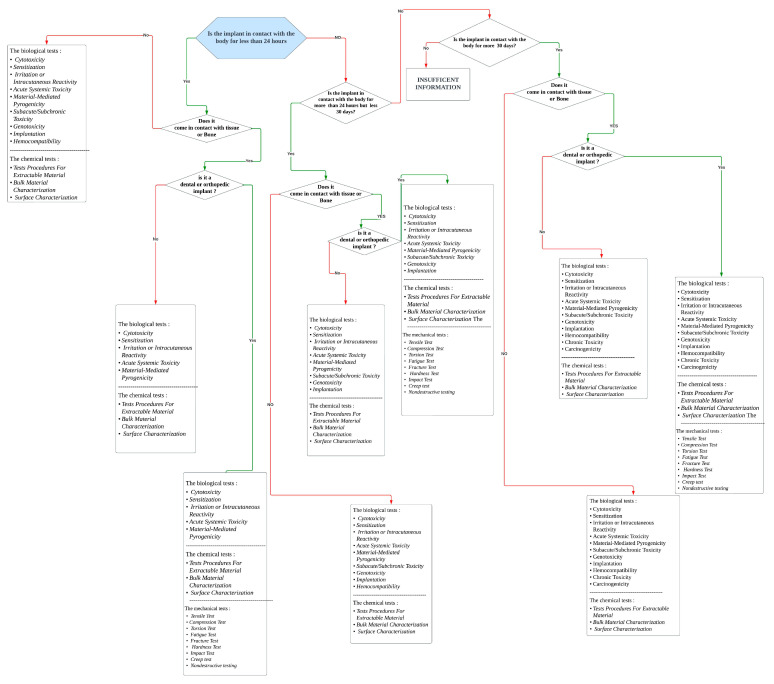
A designed flowchart with a simple “yes or no” decision-making process for manufacturers and researchers suggests a clear set of tests addressing the three major potential risk categories: mechanical, biological, and chemical.

## Data Availability

Not applicable.

## Appendix B

Available online: https://github.com/deeenabooks/BiocompPythoncode (accessed on 5 July 2023).
